# *Harpgophytum procumbens *for osteoarthritis and low back pain: A systematic review

**DOI:** 10.1186/1472-6882-4-13

**Published:** 2004-09-15

**Authors:** Joel J Gagnier, Sigrun Chrubasik, Eric Manheimer

**Affiliations:** 1 Department of Health Policy Management and Evaluation, University of Toronto, Faculty of Medicine, Toronto, Canada; 2 Canadian College of Naturopathic Medicine, Academics, Toronto, Canada; 3 Department of Forensic Medicine, University of Freiburg, Freiburg, Germany; 4 Herbal Medicines Research and Education Center, Faculty of Pharmacy, University of Sydney, Sydney, Australia; 5 Center for Integrative Medicine, University of Maryland, School of Medicine, USA

## Abstract

**Background:**

The objective of this review is to determine the effectiveness of *Harpagophytum procumbens *preparations in the treatment of various forms of musculoskeletal pain.

**Methods:**

Several databases and other sources were searched to identify randomized controlled trials, quasi-randomized controlled trials, and controlled clinical trials testing *Harpagophytum *preparations in adults suffering from pain due to osteoarthritis or low back pain.

**Results:**

Given the clinical heterogeneity and insufficient data for statistical pooling, trials were described in a narrative way, taking into consideration methodological quality scores. Twelve trials were included with six investigating osteoarthritis (two were identical trials), four low back pain, and three mixed-pain conditions.

**Conclusions:**

There is limited evidence for an ethanolic *Harpagophytum *extract containing less than <30 mg harpagoside per day in the treatment of knee and hip osteoarthritis. There is moderate evidence of effectiveness for (1) the use of a *Harpagophytum *powder at 60 mg harpagoside in the treatment of osteoarthritis of the spine, hip and knee; (2) the use of an aqueous *Harpagophytum *extract at a daily dose of 100 mg harpagoside in the treatment of acute exacerbations of chronic non-specific low back pain; and (3) the use of an aqueous extract of *Harpagophytum procumbens *at 60 mg harpagoside being non-inferior to 12.5 mg rofecoxib per day for chronic non-specific low-back pain (NSLBP) in the short term. Strong evidence exists for the use of an aqueous *Harpagophytum *extract at a daily dose equivalent of 50 mg harpagoside in the treatment of acute exacerbations of chronic NSLBP.

## Background

Natives in the steppes of South and Southwest Africa use the secondary root tubers of *Harpagophytum procumbens *(H) for the treatment of various diseases, including musculoskeletal complaints. For more than half a century, various preparations from H have been continuously used in Europe and have become an established traditional treatment for rheumatic complaints. The monograph of the European Scientific Cooperative on Phytotherapy (ESCOP) [1] recommends H preparations for painful osteoarthritis and relief of low back pain in a dosage equivalent of up to nine grams of crude plant material and over a treatment period of at least two to three months. It has been suggested that the plant material should contain not less than 1.2% of the constituent harpagoside, an iridoid glycoside. Pharmacological studies indicate that in various animal models (e.g. the writhing test in mice) [2] the extract is more effective than its marker compound harpagoside. However, a number of contradictory findings make it difficult to draw definitive conclusions on the analgesic and anti-inflammatory effect of H preparations [3].

Recent in-vitro studies indicate that preparations from H may interact with the inflammatory cascade, including the cytokines [4-6]. Moreover, a significant decrease in stimulated production of matrix-degrading enzymes has recently been shown in isolated chondrocytes [7] and a dose-dependent weak elastase inhibition [8].

The objective of this review was to determine the effectiveness of *Harpagophytum *preparations in the treatment of musculoskeletal pain.

## Methods

### Searching

Two reviewers (JG) and (SC) conducted electronic searches using the following databases: PUBMED (1966 up to September 16, 2003), EMBASE (OVID technologies: 1980 to wk 40 2003), Cochrane Controlled Trials Registry, Cochrane Musculoskeletal specialized register, Dissertation Abstracts, BIDS ISI, and the Cochrane Complementary Medicine Fields Specialized Register. The search strategy was developed by combining a highly sensitive method for isolating controlled clinical trials developed for the Cochrane Collaboration [9] [see appendix 1] with a variety of indexing and text words specific to the intervention (*H. procumbens) *and musculoskeletal conditions. Search strategies were modified for each database [for PUBMED strategy see appendix 2]. One reviewer (SC) contacted experts and acquired relevant citations. In addition, manufacturers of commercial *Harpagophytum *preparations and content experts were contacted and asked to contribute published and unpublished material. Reference lists in review articles and the retrieved trials were searched for further trials.

### Study selection

Trials that met the criteria outlined in Table 1 were included. Two individuals (JG & SC) independently reviewed titles and abstracts to determine study inclusion. A consensus method was used to resolve disagreements about inclusion of studies.

**Table 1:** Inclusion criteria for considering studies for this review

Types of studies	Randomized controlled trials (RTCs), quasi-randomized controlled trials, and controlled clinical trials (CCTs) with no language restriction.
Types of participants	Adults suffering from pain in the musculoskeletal system due to osteoarthritis or low back pain.
Types of interventions	Studies utilizing preparations of *Harpagophytum procumbens *were included. Preparations may differ in the solvent (water, alcohol) used to prepare the extract (if not crude powdered plant material is used), the drug extract ratio, and the galenic aplication form. They also differ in the content of the active principles (the sum of active ingredients) and in the quantity of the co-active marker compound harpagoside (Chrubasik et al. 1996, Sporer and Chrubasik 1999).
Types of outcome measures	Primary outcome: pain (e.g. visual analogue scale, visual rating scale, pain component of the disease-specific Arhus Low Back Pain Index, component pain of the Western Ontario MacMaster (WOMAC) instrument). Secondary outcomes: number of pain-free patients (defined as being pain-free on at least five days in the last treatment week without taking any rescue medication see above), functional indices (e.g. Lequesne index, finger-ground distance), and generic outcome measures [global assessments, health assessment questionnaire (HAQ)] or the consumption of additional analgesic treatment.

#### Data abstraction

Two reviewers (SC, JG) extracted data from each trial using a standardized form. The following data were extracted from each study: authors' names; date of publication; country of origin; type of study, including number of study centers; participants (numbers, disease(s), characteristics of the study population (age, size, weight, gender)); duration of acute exacerbation or chronic disease; baseline values with details on pain and previous treatments; additional treatments; types of outcome measures; summary statistics; timing of outcome assessment; withdrawals and drop-outs; and adverse events. Blinding to authors, institution or journal title was not done, given the contradictory evidence relating blinded reviewing and bias [10]; additionally, two reviewers (JG and SC) were very familiar with the literature.

#### Methodological quality assessment

Methodological quality was assessed using the criteria list developed by van Tulder et al (1997; 2003) [10,11]. Specifically, the internal validity criteria A, B, C, E, F, G, H, I, J, K, L1, N, and O were used. Each criterion could be scored as yes (Y), no (N), or don't know (DK). The score of Y reflects the fulfillment of that criterion. The scoring of N reflects lack of fulfillment of that criterion. The scoring of DK reflects the inability to determine whether or not the criterion was fulfilled.

High quality studies are defined as those that fulfill more than 50% (>6) of the quality criteria. Sensitivity analyses were carried out to explore the results when the definition of high quality trials was set at 40 % (>5) and 60% (>7) fulfillment of the quality criteria.

According to the Van Tulder Scoring [10,11], the levels of evidence were defined as follows:

1. *Strong *– consistent findings among multiple high quality RCTs

2. *Moderate *– consistent findings among multiple low quality RCTs and/or CCTs and/or one high quality RCT

3. *Limited *– one low quality RCT and/or CCT

4. *Conflicting *– inconsistent findings among multiple trials (RCTs and/or CCTs)

5. *No evidence *from trials – no RCTs or CCTs

Planned subgroup analyses included: (1) pain site, (2) type of pain (acute (≤ 6 weeks duration), sub-acute (6 to 12 weeks duration), and chronic pain (> 12 weeks)), and (3) comparison (botanical medicine (considering preparation form) versus placebo and botanical medicine versus other treatment).

## Results

A total of 130 citations were isolated from electronic searches and all abstracts were retrieved. A total of three additional references were supplied by content experts. A total of 120 papers were excluded because of publication type (reviews or reports) or because of improper trial design. The complete references were retrieved for the remaining 13 trials. All 13 references were included in the current review [12-24]. Of these, two trials were duplicate publications [13,21], leaving 12 trials with unique data.

A total of five randomized trials included 385 patients (range 46 to 122) with osteoarthritis of the hip or the knee [12,13,18,21-23]. Three of these trials were placebo controlled [12,18,22], and two were compared to standard pharmaceutical treatment forms [13,23]. The H preparations in these trials included a powder of crude plant material [13,22], a 60% ethanolic extract (solvent 60% ethanol)[12,18], and an aqueous extract [23].

A total of four trials included 505 patients (range 88 to 197) with acute exacerbation's of chronic non-specific low back pain [14-17]. All were randomized controlled trials, with two using placebo control [14,16], one using various conventional treatment controls (e.g. NSAIDs, exercise, massage, nerve blocks, acupuncture, etc.) [15], and one using a Cox-2 inhibitor control (Vioxx) [17]. These trials all used aqueous extracts of H.

The last three trials included 215 patients with various forms of musculoskeletal pain (range 50 to 100) [19,20,24]. Schmelz et al (1997)[24] included subjects with acute exacerbations of joint arthrosis, chronic low back pain, and rheumatic muscle pain. Guyader (1984) [20] included subjects with gonarthrosis, poly-arthrosis, coxarthrosis, and arthrosis of the cervical spine, lumbar spine, or the nerve root canal. Gobel et al (2001) [19] included subjects with pain and/or muscle tension in shoulder, neck, and/or back. All three trials were placebo controlled. Schmelz et al (1997) [24] used an aqueous extract, Guyader (1984) [20] dried mother tincture (solvent 45% ethanol), and Gobel et al (2001) [19] an ethanolic extract (solvent 60% ethanol).

All trials, except for two [12,24], reported adverse events for the interventions.

### Methodological quality and sensitivity analysis

Ratings for each trial on each quality criterion are reported in Table 2. Of the trials including patients with various forms of arthritis, four were considered high quality [13,18,22,23] and one low quality [12] with 60% and 50% cutoffs for methodological quality fulfillment; all trials were considered high quality if the cutoff was 40% [12,13,18,22,23]. There were very few instances of inadequate reporting (DK scores) in these trials (5/52) criteria assessed across trials); in cases where inadequate reporting was found, these tended not to be the same criteria across trials. The authors of these trials were not contacted for clarification regarding these items.

**Table 2:** Methodological quality of controlled trials of *Harpagophytum procumbens*

Methodological Quality Criteria	Gobel et al, 2001	Schmelz et al, 1999	Guyader, 1984	Chrubasik et al, 1996	Chrubasik et al, 1997	Chrubasik et al, 1999	Chrubasik et al, 2003	Chantre et al, 2000	Frerick et al, 2001	Lecomte & Costa, 1992	Biller et al, 2002	Schruffer, 1980
A Were eligibility criteria specified?	n	n	n	y	y	y	y	y	y	n	y	n
B Was randomization appropriate?	y	y	y	y	y	y	y	y	y	y	y	y
C Was treatment allocation concealed?	y	y	y	y	n	y	y	y	y	y	y	y
E Were groups similar at baseline regarding important prognostic indicators?	dk	dk	n	y	y	y	y	y	n	dk	dk	dk
F Were outcome measure(s) and the control interventions explicitly described?	y	y	y	y	y	y	y	y	y	y	y	y
G Were co-interventions avoided or comparable?	dk	dk	dk	y	y	y	y	y	y	dk	y	y
H Were the outcome measures relevant?	y	y	y	y	y	y	y	y	n	y	n	y
I Were adverse events described?	y	dk	y	y	y	y	y	y	y	y	n	y
J Were drop-outs described?	n	y	y	y	y	y	y	y	n	dk	n	y
K Was the sample size based on a priori power calculation?	n	n	n	y	n	y	n	y	n	n	n	n
L1 Did the study include intention-to-treat analysis? and/or	n	y	n	n	y	y	y	y	n	y	n	y
N Were point estimates and measures of variability presented for the POM?	y	n	n	y	y	y	y	y	y	y	n	n
O Was the timing of outcomes appropriate?	y	y	n	y	y	y	y	y	y	y	y	Y
**Total**	**7**	**7**	**6**	**12**	**11**	**13**	**12**	**13**	**8**	**8**	**6**	**9**

Of the trials utilizing subjects with acute exacerbations of chronic non-specific low back pain, all four were considered to be of high methodological quality regardless of the cutoff [14-17]. Methodological aspects that were unclear in the published reports were clarified by the study author (SC).

Of those trials including a mixed sample of subjects [19,20,24], two were low quality when the cutoff was 60%, or 50% and all three were high quality when the cutoff was 40%. All trials did not adequately report sufficient information to judge baseline similarity, if co-interventions were avoided or comparable, or if there were any adverse events [24].

#### *Harpagophytum *preparations for osteoarthritis

##### Powdered crude plant material compared to placebo

Lecomte & Costa (1992) [22] utilized a powder of the secondary roots of H in 89 subjects (44 placebo; 45 H) with 98 locations of arthrosis (with two locations in three subjects in the placebo group and six in the H group). Of those with one location, 31 had osteoarthritis of the spine, 18 of the cervical spine, 14 of the hip, and 30 of the knee. Results favoured the H group. Detailed descriptions of each original study included in this review are provided in Table 3.

**Table 3:** Description of trials included in this review

**Study**	**Sample Size**	**Condition; mean age (range)**	**Harpagophytum Intervention / control**	**Outcome measures and effects**	**Adverse effects**	**Reviewer's Overall Conclusions**
Schrüffler 1980 (Germany)	50	Osteoarthritis; 51 years	2500 mg/ day, (harpagoside less than 30 mg per day) / Phenylbutazone for 4 weeks	mean pain improvements: H 80%, Phenylbutazone 72%; physical impairment: H n = 1, Phenybutazone n = 5; morning stiffness: H n = 2, Phenybutazone n = 5	0 H vs 4 Phenylbutazone	H better than Phenylbutazone
Lecomte and Costa 1992 (France)	89	Osteoarthritis; (55–75) years	2000 mg/day (Harpagoside content estimated indirectly as 60 mg per day) / placebo for 60 days	mean pain improvement: H 38%, P 25% p < .05; finger-ground distance modified Schober test (cm) mean improvement: H 16%, P 6% p < .05	none for either group	H better than placebo
Biller 2002 (Germany)	78	Osteoarthritis; not stated	4500 mg/day, (harpagoside content estimated at < 30 mg per day) / placebo for 20 weeks	responders: H 90%, P 80% p-value not stated; mean consumption of ibuprofen: H .1, P .5 tablets	not stated	H better than placebo
Chantre et al. 2000 (France)	122	Osteoarthritis; 62 years	4500 mg / day, (57 mg harpagoside per day) / Diacerhein for 16 weeks	difference after 16 weeks between groups as measured by Lequesne functional index: less than 10 mm NS (intention-to-treat analysis with not all possible confounders considered)	10 H vs 21 Diacerhein	H not worse than diacerhein
Frerick et al. 2001 (Germany)	46	Osteoarthritis; 59 years	4500 mg/day, (< 30 mg harpagoside per day) / placebo for 20 weeks	responders: H 71%, P 41% p=.041; WOMAC component pain NS (type of statistical analysis not stated)	8 H vs 7 P	H better than placebo
Chrubasik et al. 1996b (Germany)	118	Back pain; 54 years	4500 mg/day, (50 mg harpagoside per day) / placebo for 4 weeks	mean tramadol consumption: H 99 ± 157 mg, P 102 ± 250 mg p =.44; number of pain-free patients at 4th week: H 9 P 1 p=.008; percentage change Arhus component pain: H 34%, P 6% p=.016 (per protocol analysis)	4 H vs 10 P	not on primary outcome measure
Chrubasik et al. 1997 (Germany)	102	Back pain; 49 years	4500 mg/day, (30 mg harpagoside per day) / conventionally treating physicians administering oral NSAIDs, physical exercises, or paravertaebral injections for 6 weeks	number of pain-free patients 4th week: H 16, C12 NS; number of pain free patients 6th week: H 20, C 23 NS; percentage change Arhus component pain after four weeks: H 23%, C 22% p=.95; after 6 weeks H 33%, C 38% p=.38	5 H vs 0 C	H not worse than C
Chrubasik et al. 1999 (Germany)	197	Back pain; 56 years	4500 and 9000 mg/day, (50 and 100 mg harpagoside per day) / placebo for 4 weeks	number of pain-free patients: H-100 18%, H-50 9%, P 5% p=.027); percentage change Arhus component pain: H-100 vs H-50 vs P NS (intention-to-treat analysis)	10 P, 18 H-50, 17 H-100	H better than placebo
Chrubasik et al. 2003a (Germany)	88	Back pain; 62 years	4500 mg/day, (60 mg harpagoside per day) / Rofecoxib for 6 weeks	number of pain-free patients: H 22%, Rofecoxib 11% NS; percentage change Arhus component pain: H 30%, Rofecoxib 29% (intention-to-treat analysis)	14 H, 14 Rofecoxib	H not worse than Rofecoxib
Schmelz and Hämmerle 1999 (Germany)	100	Mixed pain; not stated	4500 mg/day, (30 mg harpagoside per day) / placebo for 30 days	free of low back pain: H n = 4, P n = 2; free of other pain: H n = 5, P n = 0 (confounders not considered)	not stated	H better than placebo
Guyader 1984 (France)	50	Mixed pain; 64 years	Harpagoside content estimated indirectly as <20 mg harpagoside per day / placebo for 1–3 'cycles' of 21 days each	mean pain improvements: H 72%, P 65% (confounders not considered)	6 H vs 3 P	H better than placebo
Goebel et al. 2001 (Germany)	65	Mixed pain; 28 years	4500 mg/day, (< 30 mg harpagoside per day) / placebo for 28 days		4 H vs 2 P	H better than placebo

Key: NS = not significant; H = harpagophytum; P = placebo; WOMAC = Western Ontario and McMaster Universities Arthritis Index

##### Powdered crude plant material compared to diacerhein

Chantre et al (2000) [13] gave H cryodried drug powder (proprietary product Harpadol^R^) or Diacerhein (D), to 122 subjects with acute exacerbations of hip and knee. Groups did not differ significantly in sponanteous pain or the Lequesne index, though differences from baseline were larger for the H group. Subjects in the H group used less diclofenac (mean = 21 tablets) than those in the D group (60 tablets) and also used less acetominophen-caffeine (H = 40 tablets and D = 60 tablets).

##### H extract (solvent 60% ethanol) compared to placebo

Biller (2002) [12] gave knee arthrosis participants a H product named Flexiloges^R ^or placebo in addition to ibuprofen (at 800 mg ibuprofen during weeks 1–8, 400 mg during weeks 9 – 16), and only H or placebo during weeks 17–20. The main outcome measure was the responder rate, which allowed a WOMAC pain score increase of up to 20% and no additional consumption of ibuprofen in weeks 17 to 20. Results favoured the H group.

Frerick et al (2001) [18] gave 46 individuals with acute exacerbations of coxarthrosis Flexiloges^R ^or placebo in addition to ibuprofen (at 800 mg ibuprofen during weeks 1–8, 400 mg during weeks 9–16) and only H or placebo during weeks 17–20. The main outcome was the responder rate, which was defined as the number of patients that required fewer than 4000 mg ibuprofen and had a pain score increase of no more than 20% on the WOMAC component pain during weeks 17 to 20. Results indicated more responders in the H group.

##### Aqueous extract compared to NSAID

Schruffler (1980) [23] compared H (proprietary product, Salus^R,^) with phenylbutazone among 40 individuals with acute exacerbations of rheumatic joint and muscle pain and 10 with gouty arthritis. Results favoured the H group.

#### *Harpagophytum procumbens *for acute exacerbation of chronic non-specific low back pain (NSLBP)

##### Aqueous extract compared to placebo

In the Chrubasik study (1996) [14], 128 patients suffering from pseudo-radiating or non-radiating NSLBP were allocated to receive either a proprietary extract, Doloteffin^R,^, or placebo. Results favoured the H group (see Table 4). Of the 59 patients in the H group, five dropped out, one of these due to tachycardia. Of the 59 patients in the P group, four dropped out for unknown reasons. A total of four adverse effects occurred in the H group. These consisted of two individuals with nausea/emesis due to the tramadol, one patient with repeated tussive irritation, and the patient with tachycardia mentioned above. A total of 10 adverse events occurred in the P group. These included nausea (N = 2), and one patient each of nausea/vertigo due to tramadol, fatigue/vertigo, vertigo alone, diuresis/normalization of constipation (i.e. intractable constipation), constipation (several times), diuresis (several times), and sleep disturbances (permanent).

An additional study by Chrubasik et al (1999) [16] randomized subjects suffering from pseudo-radiating or non-radiating NSLBP were allocated to receive the proprietary extract WS1531, at a dose equivalent to either 4500 mg H (with 50 mg harpagoside per day, H_50_) or 9000 mg H /day (with 100 mg harpagoside per day, H_100_), or placebo (P; N = 66). Participants had acute exacerbations of non-specific low back pain; current pain that was > 5 on a VAS (0–10). The median durations of chronic pain were P 15 years, H_50 _15 years, H_100 _15 years. The number of patients with acute exacerbations of greater than 3 months for each group was P 54 (82%), H_50 _53 (82%), H_100 _55 (83%). Current pain and Arhus scores were similar among all groups. The principle outcome measure, the number of patients who were pain-free without the permitted rescue medication for 5 days out of the last treatment week was 3 (P), 6 (H_50_) and 10 (H_100_) (p = 0.027, one-tailed Cochrane-Armitage test). The authors found significant improvements in pain in both H groups as compared with the placebo group. A subgroup analysis found differences between groups for those without pain radiating to the legs (H_100 _40%, H_50 _43% and P 23%; P = 0.017) and for those without a neurological deficit (H_100 _40%, H_50 _60% and P 20%; P = 0.034).

##### Aqueous extract compared to NSAID

Chrubasik et al (2003) [17] randomized 88 individuals suffering from pseudo-radiating or non-radiating NSLBP recieved either the proprietary extract Doloteffin^R ^(N = 44) or Vioxx^R ^(rofecoxib; R; N = 44). The percentage of subjects with chronic pain of > 6 days for each group was H 84%, R 84% and acute pain of > 90 days in H 91%, R 89%. Results indicated statistically non-significant difference between H and R. Also, a total of 21 (group H) and 13 (group R) patients used tramadol, with the average consumption being 230 mg (H) and 133 mg (R). A total of seven drop-outs occurred due to adverse events (H = 1; R = 6); two others resulted from excessive low back pain (R = 2). There were a total of seven protocol violations. A total of 14 participants in each group had adverse events with gastrointenstinal complaints, equaling eight in the H group and nine in the R group, with more severe events in the R group. Two adverse events were reported to be unrelated to the study medication in the H group.

##### Aqueous extract compared to various conventional treatments

In another study by Chrubasik et al (1997) [15], participants suffering from non-radiating NSLBP were randomized to receive either the proprietary extract Jurcuba^R ^or conventional treatments (NSAIDs, exercise, massage, nerve blocks, acupuncture). Subjects' pain was > 5 (VAS 0–10) on at least two of the following five scores: pain at rest, while sitting, lying, and walking or at night. The median duration of acute pain was six weeks for both groups. The median duration of chronic pain was 120 months for the H group and 72 months for the control group. There were no statistically significant differences between the groups.

#### *Harpagophytum *preparations for mixed pain conditions

##### Dried mother tincture compared to placebo

In a placebo controlled double-blind study, Guyader (1984) [20] included 50 patients with poly-arthrosis (N = 14), coxarthrosis (N = 2), arthrosis of the cervical spine (N = 11), lumbar spine (N = 2), and arthrosis of the nerve root canal (N = 6). Subjects were given either "Extract G", a dried mother tincture of Devil's claw (H), or placebo (P) for one to three cycles (18 single P, 16 single H, 6 PH, 4 HP, 2 HH, 1 PPP, 1 PHH, 1 PPH, 1 PHP) of 21 days with seven day intervals. Outcome measures included pain at rest, pain during exercise, joint pressure pain, pain while walking (in cases of cox- and gonarthrosis), and pain at night. All outcomes were taken 10 days after each cycle and assessed on a five-point rating scale for amount of pain (no pain = 0, mild = 1, moderate = 2, severe = 3, excrutiating = 4). A total of 70 cycles were analyzed (37 cycles for P and 33 for H) and a mean pain improvement of 72% in the H group and 65% in the placebo group (p < 0.05) was found. Drop-outs included two subjects. During the H cycles, six adverse events occurred, including nausea, gastralgia, diarrhea, severe constipation, pruritic eruptions with erythema, and generalized pruritis. During the P cycles, five adverse events were observed, including gastralgia, sweating, headache, and aerophagy.

##### Aqueous extract compared to placebo

In a double-blind, placebo-controlled trial, Schmelz et al (1997) [24] randomized 100 individuals with acute exacerbations of joint arthrosis (N = 29 (H), N = 27 (P)), chronic low back pain (N = 14 H, N = 17 P), and rheumatic muscle pain (N = 7 H, N = 6 P). Subjects were given either the proprietary extract Arthrotabs^R ^containing extract based on 4500 mg crude plant material (equivalent of 30 mg harpagoside) per day or a placebo for 30 days. Outcome measures included a subjective pain scale (no pain, mild pain, moderate pain, severe pain, excrutiating pain) at baseline and after four weeks of treatment. The number of pain-free individuals with low back pain was H n = 4, P n = 2, and for other pain sites H n = 5, P n = 0.

##### *Harpagophytum *extract (solvent 60% ethanol) compared to placebo

Gobel et al (2001) [19] conducted a double-blind study in 65 individuals with pain or muscle tenseness in shoulder, neck, and/or back having lasted for 14 days prior to the study. They were randomized to placebo (N = 32) or a proprietary extract (Rivoltan; N = 31). The daily extract dosage was based on 4500 mg crude plant material (equivalent to <30 mg harpagoside per day). Outcomes included a visual analogue scale for pain (0–50 mm) as well as an experimental test battery for pain and muscle tension before and after treatment. In the per-protocol analysis the Hgroup had less pain than the P group. A total of two individuals dropped out of the trial with four adverse events occurring in the H group versus two in the P group.

## Discussion

Osteoarthritis of the knee, hip, and spine as well as non-specific low back pain may be associated with pain, stiffness, limitation of function, and diminished quality of life [25]. Although treatment guidelines recommend simple analgesics as first-line drugs [26], surveys indicate that NSAIDs are used in preference to simple analgesics despite the lower safety-margin and the higher cost [11,27,28]. Because of the high incidence of NSAID-related adverse events and complications in the gastrointestinal and cardiovascular systems (especially in the elderly), and the high costs related to adverse events (i.e. gastrointestinal bleeding or perforation), additional medical attendances, diagnostic procedures, treatments and admissions to hospital, alternatives to NSAID therapy should be strongly considered [29-33].

This qualitative analysis of the 12 trials suggests that specific preparations and doses of *Harpagophytum procumbens *may be effective in various types of musculoskeletal pain conditions. Statistical pooling was not possible because of a lack of adequate data and clinical heterogeneity. The sensitivity analysis for methodological quality revealed that the trials on low back pain were of high quality, the trials on osteoarthritis were of high quality except one moderate quality study, and the trials on mixed pain conditions were of moderate quality. The quality of reporting in most of these trials was good. In order to increase transparency, trialists should refer to the CONSORT statement in designing and reporting clinical trials of herbal medicinal products [34].

One high quality trial indicates that there is moderate evidence of effectiveness for powdered H plant material at a dose equivalent to 60 mg of harpagoside per day for osteoarthritis of the spine, hip, and knee. However, because of the clinical heterogeneity of patients in this trial, a confirmatory study is required to firmly establish efficacy for each location of osteoarthritis. In one high quality study, 4500 mg powder containing 57 mg harpagoside in the daily dosage showed moderate evidence for non-inferiority to diacerhein in patients suffering from acute exacerbations of osteoarthritis in the hip and knee.

Two trials employed an ethanolic extract (solvent 60% ethanol) containing less than 30 mg harpagoside per day in patients with osteoarthritis of the knee [12] and hip [18]. Both trials showed statisitically significant favourable results for the H group in terms of percentage of responders. However, the definition of responder in these trials may be questioned because of an allowance of pain increase up to 20% and additional rescue medication in one of the studies [18]. Therefore, given the low methodological quality of the trials and the lack of clinically significant differences between groups, we conclude there is limited evidence for the use of an ethanolic H extract based on 4500 mg crude plant material per day in patients with osteoarthritis of the knee and hip. Additional high quality trials must be done to determine the efficacy of *Harpagophytum procumbens *in osteoathritis. These trials must include homogenous pain conditions and must test H against standard osteoarthitis medications. Additionally, trialists should consider using symptom severity outcome measures that have proven validity and reliability, such as visual analogue scales [35-37], osteoarthritis specific outcome measures (e.g. WOMAC, Lequesne Index) [35-38], and health-related quality of life instruments (e.g. Medical Outcomes Survey Short-Form 36) [35,37,38].

A total of four high quality trials tested various dosages of Hextract in acute exacerbations of chronic non-specific low back pain. Two trials with a total of 325 patients showed that an aqueous extract at the equivalent daily dosage of 50 mg harpagoside appears to reduce pain in patients with acute episodes of chronic NSLBP greater than does placebo [14,16]. Therefore, the 50 mg harpagoside per dose of an aqueous extract of H can be said to have strong evidence for the treatment of acute episodes of chronic NSLBP in the short term. Additionally, a one year survey indicates that the aqueous extract is well tolerated [38]. One trial with 197 patients showed that an aqueous H extract at the equivalent daily dose of 100 mg harpagoside appears to reduce pain in patients with acute episodes of chronic NSLBP greater than does placebo [16]. Therefore, the 100 mg harpagoside per dose of an aqueous H extract has moderate evidence for the treatment of acute episodes of chronic NSLBP in the short term. Superiority of the higher dose was seen in the primary outcome (number of pain-free patients) but not in the secondary outcome measure. Therefore, there is moderate evidence for superiority of the 100 mg H dose to the 50 mg H dose. However, additional trials are required to confirm superiority of 100 mg H over 50 mg H. It is possible that a subgroup of individuals with neurological deficits (e.g. radiation into the leg) may respond well to the 100 mg harpagoside dose, yet more research is required to clarify this.

An aqueous extract of H at the equivalent daily dose of 60 mg harpagoside appears to be equivalent to 12.5 mg Rofecoxib in improving pain in individuals with acute episodes of chronicNSLBP [17]. Therefore, a 60 mg daily harpagoside dose in aqueous extract of H has moderate evidence for being not inferior to 12.5 mg rofecoxib per day in the treatment of acute episodes of chronic NSLBP in the short term. Additional high quality trials, especially over longer treatment periods, are mandatory. Furthermore, equivalence trials testing *Harpagophytum procumbens *against standard treatments will clarify relative efficacy and safety.

The final trials (two of moderate [19,24] and of poor quality [20] included heterogeneous musculoskeletal pain conditions. Therefore, it is difficult to reach any conclusions on the basis of these trials. Future trials should attempt to include homogenous pain conditions.

The results obtained with the proprietary *Harpagophytum *products containing aqueous extracts can neither be transferred to an aqueous extract containing less harpagoside in the daily dosage [41] or to a product containing an ethanolic extract. This can only be done if the ethanolic extract was shown to be essentially similar to aqueous extract, and if both extracts have the same qualitative and quantitative composition of co-active constituents, same pharmaceutical form, and bioequivalence in terms of safety and efficacy [40]. With 60% ethanol as solvent, only half the amount of harpagoside (and possibly other co-active constituents) is extracted compared to water as solvent [42]; therefore, it is of great importance that a confirmatory study provide evidence of effectiveness for the ethanolic extract.

Since the "active principle" has not yet been identified for *Harpagophytum procumbens, *the constituent harpagoside is used as a marker for standardization of *Harpagophytum *preparations. For harpagoside, the dose-dependent absorption into systemic circulation has been shown and may be related to lipoxygenase inhibition [43]. However, it remains to be established if the inhibitory effect on leukotriene production corresponds to therapeutic efficacy. Future research should attempt to identify the active constituent or profile of constituents that relate to therapeutic efficacy in order to make extract dosing transparent.

There are several drawbacks to the present study. First, this is a qualitative review and as such it does not provide a quantitative summary of results, thus making it difficult to determine the size of effect of each intervention. Secondly, this review includes a small number of trials, often with small sample sizes. This makes it difficult to state definitive conclusions of efficacy and suggests the need for more trials. On the other hand, the trials reviewed were generally of good methodological quality and have several statistically significant and clinically significant effects. Therefore, these trials help us reach some clear conclusions regarding the use of specific preparations and doses of *Harpagophytum procumbens *for osteoarthritis and non-specific low back pain. Another strength of this study is the comprehensive search strategy, the methodological quality assessment, and the use of an accepted method for a best evidence synthesis. Future reviews may attempt to statistically combine the results of such trials into a meta-analysis.

## Conclusions

There is limited evidence for an ethanolic *Harpagophytum *extract containing less than <30 mg harpagoside per day in the treatment of knee and hip osteoarthritis. There is moderate evidence of effectiveness for (1) the use of a *Harpagophytum *powder at 60 mg harpagoside in the treatment of osteoarthritis of the spine, hip and knee; (2) the use of an aqueous *Harpagophytum *extract at a daily dose of 100 mg harpagoside in the treatment of acute exacerbations of chronic non-specific low back pain; and (3) the use of an aqueous extract of *Harpagophytum procumbens *at 60 mg harpagoside being non-inferior to 12.5 mg rofecoxib per day for chronic non-specific low back pain (NSLBP) in the short term. Strong evidence exists for the use of an aqueous *Harpagophytum *extract at a daily dose equivalent of 50 mg harpagoside in the treatment of acute exacerbations of chronic NSLBP.

## Competing interests

One individual (SC) was an author of several original trials included in this systematic review. This did not appear to influence the content of this paper.

## Authors' contributions

JG developed the initial idea for the manuscript, searched for trials, extracted data and wrote and edited the manuscript.

SC developed the initial idea for the manuscript, searched for trials, extracted data, and wrote and edited the manuscript.

EM extracted data and wrote and edited the manuscript.

## Appendix 1

Highly sensitive search strategy for randomized controlled trial searches using PUBMED

(randomized controlled trial [pt] OR controlled clinical trial [pt] OR randomized controlled trials [mh] OR random allocation [mh] OR double-blind method [mh] OR single-blind method [mh] OR clinical trial [pt] OR clinical trials [mh] OR (clinical trial [tw]) OR ((singl* [tw] OR doubl* [tw] OR trebl* [tw] OR tripl* [tw]) AND (mask* [tw] OR blind* {tw])) OR (latin square [tw]) OR placebos [mh] OR placebo* [tw] OR random* [tw] OR research design [mh:noexp] OR comparative study [mh] OR evaluation studies [mh] OR follow-up studies [mh] OR prospective studies [mh] OR cross-over studies [mh] OR control* [tw] OR prospectiv* [tw] or volunteer* [tw]) NOT (animal [mh] NOT human [mh])

## Appendix 2

PUBMED search strategy

(randomized controlled trial [pt] OR controlled clinical trial [pt] OR randomized controlled trials [mh] OR random allocation [mh] OR double-blind method [mh] OR single-blind method [mh] OR clinical trial [pt] OR clinical trials [mh] OR (clinical trial [tw]) OR ((singl* [tw] OR doubl* [tw] OR trebl* [tw] OR tripl* [tw]) AND (mask* [tw] OR blind* {tw])) OR (latin square [tw]) OR placebos [mh] OR placebo* [tw] OR random* [tw] OR research design [mh:noexp] OR comparative study [mh] OR evaluation studies [mh] OR follow-up studies [mh] OR prospective studies [mh] OR cross-over studies [mh] OR control* [tw] OR prospectiv* [tw] or volunteer* [tw]) NOT (animal [mh] NOT human [mh]) AND ("harpagophytum procumbens" OR (devil's AND claw)) AND (pain OR "musculoskeletal pain" OR "muscle pain" OR "skeletal pain" OR "bone pain" OR "joint pain" OR "extremity pain" OR myaligia OR osteoarthritis OR "rheumatoid arthritis" OR arthrosis OR "low back pain" OR lumbago OR "back pain") NOT review

## Pre-publication history

The pre-publication history for this paper can be accessed here:


